# *Klebsiella pneumoniae* invasive syndrome with liver, lung, and brain abscesses complicated with pulmonary fungal infection: a case report and review of the literature

**DOI:** 10.1186/s12245-023-00574-1

**Published:** 2023-12-21

**Authors:** Yunhao Luo, Wen Hu, Lingna Wu, Shijie Duan, Xingmei Zhong

**Affiliations:** 1 Department of Critical Care Medicine, Chengdu First People’s Hospital, Chengdu, China; 2 Department of Critical Care Medicine, Chengdu Seventh People’s Hospital, Chengdu, China

**Keywords:** *Klebsiella pneumoniae* invasion syndrome, Liver abscess, Brain abscess, Lung abscess, Pulmonary fungal infection, Case report

## Abstract

**Background:**

*Klebsiella pneumoniae* invasion syndrome (KPIS) is a severe multi-site infection that is usually caused by hypervirulent *Klebsiella pneumoniae*. The bacteria are relatively common in Asian diabetics and can cause organ abscesses or sepsis. When patients develop intracranial infection, the prognosis is poor. After anti-infective treatment, the *Klebsiella pneumoniae*-induced liver and lung abscesses and pulmonary fungal infection were relieved, but the brain abscesses worsened. Such complex and severe infection cases are rarely reported. Early identification of intracranial infection, selection of antibiotics with high concentrations in cerebrospinal fluid, and active treatment of complications such as diabetes and fungal infection are of great significance for the prognosis of patients.

**Case presentation:**

A 71-year-old patient diagnosed with liver abscess in another hospital was transferred to our hospital due to a worsening condition. On day 1 (day of admission), the patient was given invasive mechanical ventilation, continuous renal replacement therapy combined with endotoxin adsorption, antimicrobial treatment with imipenem-cilastatin, and percutaneous catheter drainage for liver abscess. Metagenomic next-generation sequencing in bronchoalveolar lavage fluid indicated *Klebsiella pneumoniae (K. pneumoniae)*, *Candida albicans*, and *Aspergillus flavus complex*, and no viruses were detected. Blood and pus cultures revealed *K. pneumoniae* that was sensitive to piperacillin/tazobactam. The anti-infection therapy was adjusted to piperacillin/tazobactam combined with voriconazole. On day 14, a head computed tomography (CT) scan showed no significant changes, and a chest CT scan showed absorption of multiple abscesses in both lungs. The patient was still unconscious. After the endotracheal tube was removed, cranial magnetic resonance imaging (MRI) showed multiple brain abscesses. Finally, his family gave up, and the patient was discharged and died in a local hospital.

**Conclusion:**

In cases of *K. pneumoniae* infection, the possibility of intracranial, liver, lung, or other site infections should be considered, and physicians should be vigilant for the occurrence of KPIS. For patients suspected of developing an intracranial infection, cerebrospinal fluid should be tested and cultured as soon as possible, a head MRI should be performed, and antibiotics with high distribution in cerebrospinal fluid should be used early. When patients are complicated with diabetes, in addition to glycemic control, vigilance for concurrent fungal infections is also needed.

## Introduction

Liver abscess is one of the most common visceral abscesses. The risk factors include diabetes, hepatobiliary, and pancreatic diseases. Gram-negative bacilli such as *Escherichia coli* and *Klebsiella pneumoniae* are the main pathogens associated with liver abscesses [1]. *K. pneumoniae* was more common in Asian patients than in non-Asian patients (50% vs 27%, respectively) [2]. In a recent meta-analysis, diabetes and liver abscess (pooled risk ratio: 2.61 and 9.04; all *P* < 0.001) were predictors of hypervirulent *K. pneumoniae* (hvKp) infections [3]. Patients infected with hvKp or with poor glycemic control of diabetes may progress to KPIS with multiple abscesses of the liver, lung, brain, and other sites [4, 5]. It has been reported that the mortality of *K. pneumoniae* meningitis is 53% [6], and the mortality is significantly increased when multiple abscesses, such as those in the liver and brain, occur at the same time [7]. Fungal infection also increases the risk of death [8]. In this case report, we share a rare case of KPIS with the liver, lung, and brain abscesses complicated with pulmonary fungal infection in a diabetic patient with poor glycemic control.

## Case presentation

A 71-year-old man presented with fever and dyspnea for 3 days. His highest temperature was 39.5°C, accompanied by dyspnea, nausea, and vomiting. The patient also experienced dizziness and fatigue, with no headache, chest pain, abdominal pain, altered consciousness, etc. One day before admission, the patient visited a local hospital. Laboratory investigations revealed a white blood cell (WBC) count of 13.97 *10^9/L [reference range: 3.5–9.5 *10^9/L], neutrophil percentage of 91.8% [reference range: 40–75%], platelet (PLT) of 54 *10^9/L [reference range: 100–300 *10^9/L], and CRP of 313.96 mg/L [reference range: < 10 mg/L]; hypersensitive C-reactive protein (hs-CRP), > 10 mg/L [reference range: < 10 mg/L], and procalcitonin (PCT), > 95 ng/ml [reference range: < 0.5 ng/ml]. The head CT scan showed brain atrophy. The chest CT scan revealed bilateral pulmonary nodules, with the larger nodule measuring approximately 7*5 mm. There was minimal pleural effusion bilaterally. The abdominal CT scan indicated a round-shaped slightly hypodense lesion with a diameter of approximately 46 mm at the top of the right lobe of the liver, possibly a liver abscess. Because these CT scans were performed at another hospital, we only have textual reports and do not have images. The main diagnoses were sepsis, acute respiratory distress syndrome, and liver abscess at the local hospital. The patient received non-invasive mechanical ventilation and was treated with imipenem-cilastatin. Due to the worsening of the patient’s condition and progressive alteration of consciousness, he was transferred to our hospital. Endotracheal intubation was performed in the emergency department of our hospital, and the patient was admitted to the intensive care unit. The patient’s conscious state was coma, with a Glasgow Coma Scale (GCS) score of 5. Auscultation of the chest revealed moist rales and no pain expression when pressing the abdomen. Meningeal irritation signs and pathological signs were negative.

**Fig. 1:**
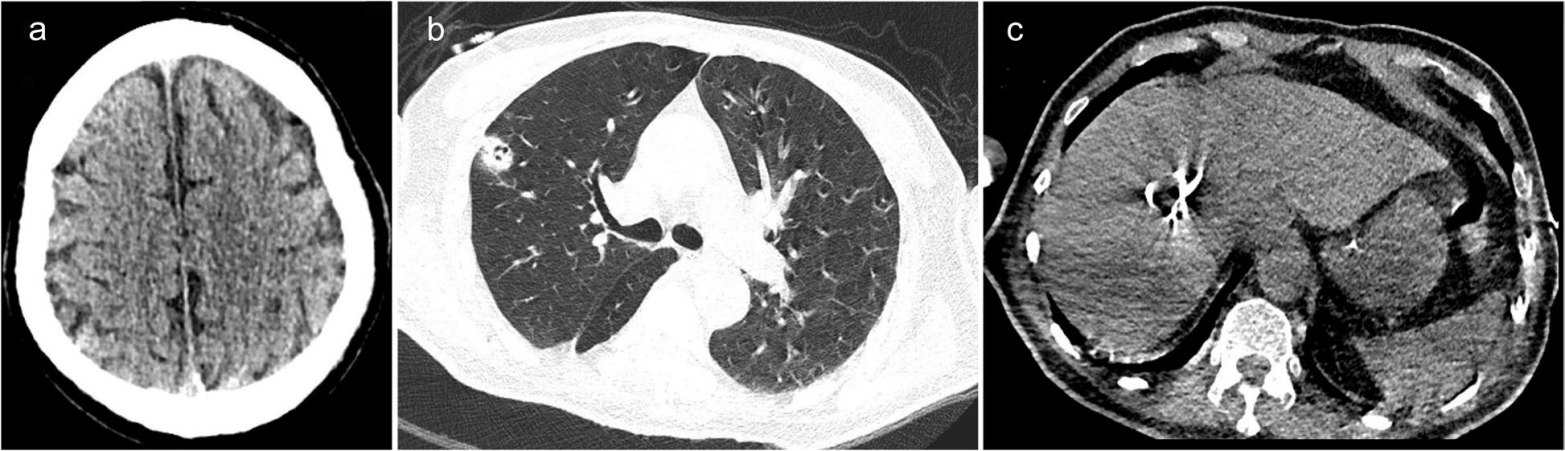
On day 7, the head CT scan showed hypodense lesions around bilateral frontal lobes, basal ganglia, and lateral ventricles, suggestive of lacunar cerebral infarctions (**a**). The chest CT scan showed bilateral pulmonary nodules with central cavitation. The largest nodule was located in the right upper lobe and measured approximately 23*17 mm (**b**). The abdominal CT scan revealed an abscess in the right lobe of the liver, with a maximum cross-section of 33.69*37.21 mm and catheter signs (**c**)

**Fig. 2:**
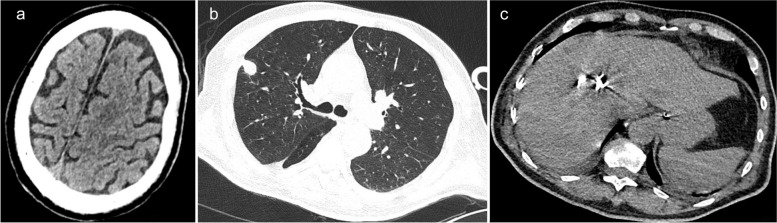
On day 14, the head CT scan showed hypodense lesions, with no apparent changes compared to previous findings (**a**). The chest CT scan revealed that bilateral pneumonia and abscesses had been absorbed compared to previous findings (**b**). The abdominal CT scan showed a reduction in the size of the hepatic abscess in the right lobe of the liver compared to previous findings, with the presence of features indicative of a catheter within it (**c**)

**Fig. 3:**
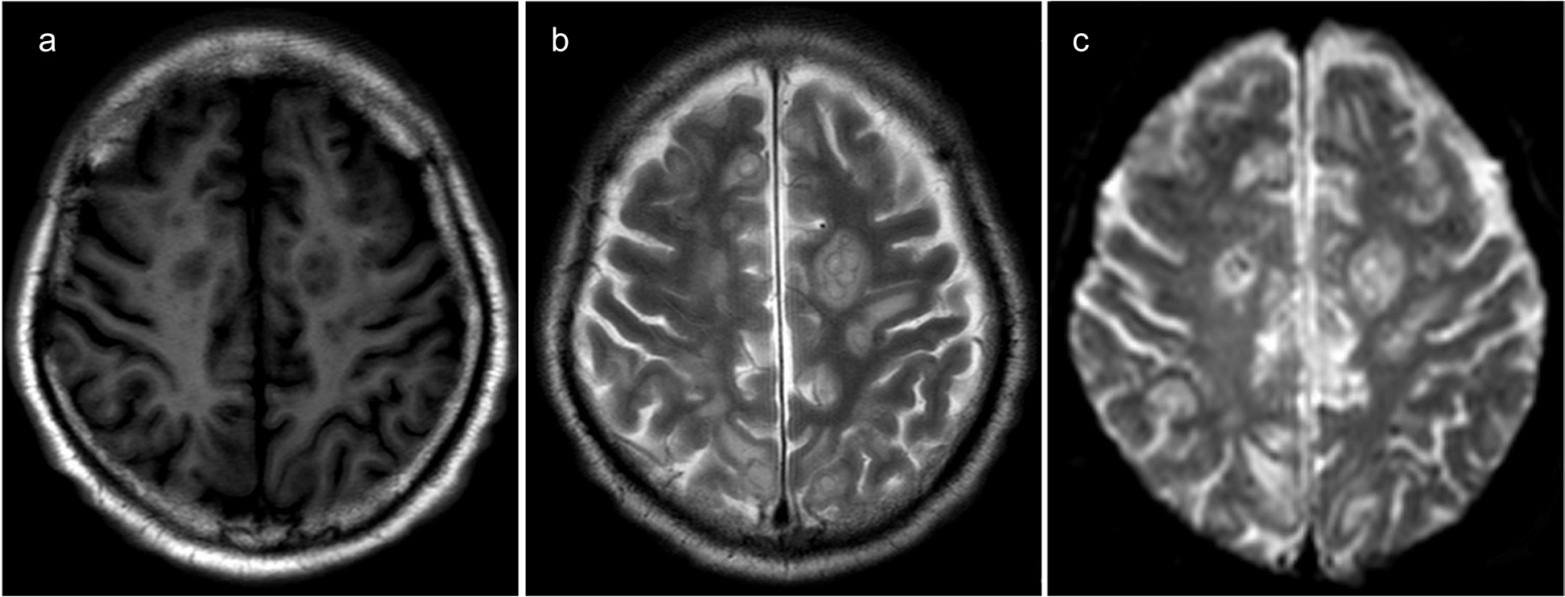
On day 21, the brain magnetic resonance imaging (MRI) revealed widespread plaques or circular abnormal signals within the brain parenchyma. These signals appeared as low intensity on T1-weighted imaging (**a**), high intensity on T2-weighted imaging (**b**), and showed restricted diffusion on diffusion-weighted imaging (**c**)

After admission, the patient received invasive mechanical ventilation, sedation, and analgesia. Laboratory results showed WBC, 16.75 *10^9/L; neutrophil ratio, 90.1%; PLT, 54 *10^9/L; hs-CRP, 268.48 mg/L; PCT, > 95 ng/mL; aspartate aminotransferase (AST), 169 U/L [reference range: 0–50 IU/L]; alanine aminotransferase (ALT), 86 U/L [reference range: 0–50 IU/L]; venous blood glucose, 29.02 mmol/L [reference range: 3.9–6.1 mmol/L]; creatinine, 142 µmol/L [reference range: 45–84 µmol/L]; and urea nitrogen, 10.48 mmol/L [reference range: 2.1–7.2 mmol/L] (Table 1). Color Doppler ultrasound indicated a mixed echogenic mass of 4.4*4.8 cm in segment 8 of the liver, which was considered an abscess. Due to incomplete liquefaction of the liver abscess, drainage was not performed at this time. Clinical pharmacists recommended that the patient continued to use imipenem-cilastatin (1g i.v. q6h, infusion time > 3h). At the same time, he received liver protection, expectoration, nutritional support, and glycemic control. On day 3, the patient developed septic shock, and norepinephrine was administered to raise his blood pressure. He also developed decreased urine output and acute kidney injury, so continuous renal replacement therapy combined with endotoxin adsorption was given. The patient’s routine blood test showed a very low PLT of 31 *10E9/L (Table 1), and percutaneous catheter drainage was intended, so 1 unit of platelets was transfused. On day 7, the patient’s WBC increased to 34.79 *10^9/L, neutrophil ratio was 80.4%, and hs-CRP was 97.85 mg/L (Table 1). His activated partial thromboplastin time was 162.8s, and 400ml of fresh frozen plasma was transfused. The (1,3)-beta-d-glucan antigen test result was 137.5pg/ml [reference range: 0–70 pg/ml]. Galactomannan test result was normal. Metagenomic next-generation sequencing in bronchoalveolar lavage fluid indicated *K. pneumoniae*, *Candida albicans*, and *Aspergillus flavus complex*, and no viruses were detected. Blood culture results showed *K. pneumoniae* (extended-spectrum β-lactamase negative, sensitive to piperacillin/tazobactam, tetracyclines, macrolides, cephalosporins, aminoglycosides, quinolones, carbapenems; resistant to ampicillin and ticacillin). Considering that the patient had developed acute kidney injury, the antibiotic was adjusted to piperacillin/tazobactam (4.5g i.v. q8h) combined with voriconazole (200mg i.v. q12h, 300mg i.v. q12h on the first day). Color Doppler ultrasonography showed that the liver abscess turned into a mixed sac mass of 6.4 × 5.3 cm. He underwent ultrasound-guided percutaneous catheter drainage of the liver abscess. The pus was pale red and was sent for microbial culture. After discontinuation of analgesic and sedative medications, he remained in a coma with a GCS score of 4. The head CT scan showed lacunar cerebral infarction (Fig. 1a). The chest CT showed pneumonia and pulmonary nodules with cavities (Fig. 1b). The abdominal CT scan revealed an abscess in the right lobe of the liver and catheter signs (Fig. 1c). On day 10, pus culture results also showed *K. pneumoniae* that was sensitive to piperacillin/tazobactam. On day 14, the reexamination of the head CT scan showed no significant changes compared with before (Fig. 2a). The reexamination of the chest CT scan showed that bilateral pneumonia and abscesses were absorbed more than before (Fig. 2b). The reexamination of the abdominal CT scan showed a reduction in the size of the hepatic abscess in the right lobe of the liver compared to previous findings (Fig. 2c). On day 15, there was no liquid extraction from the drainage tube of the liver abscess, and there was no obvious liquid area in the reexamination of color Doppler ultrasound, so the drainage tube was removed. The patient’s lung abscesses and liver abscess were relieved, and the endotracheal tube was removed, but he remained in a coma with a GCS score of 3. On day 21, a cranial MRI showed multiple brain abscesses (Fig. 3a–c). The neurologist recommended a lumbar puncture, but the family refused. The final diagnoses were KPIS, pulmonary fungal infection, multiple organ failure, and sepsis. Finally, the patient’s family gave up and discharged him. The patient died at a local hospital 3 days after being discharged from our hospital.

**Table 1:** Laboratory test results

Date	WBC *10^9/L	Neutrophil percentage %	PLT *10^9/L	hs-CRP mg/L	PCT ng/mL	AST U/L	ALT U/L	Creatinine umol/L	Urea nitrogen mmol/L
Day 1	16.75	90.1	54	268.48	no	169	86	142	10.48
Day 2	12.95	87.1	47	282.49	> 95.00	160	80	139	12.42
Day 3	17.07	92	31	273.09	/	183	77	293	19.75
Day 4	14.87	85.4	45	260.41	88.5	/	/	/	/
Day 5	15.13	74.5	45	166.87	/	69	40	128	13.62
Day 7	34.79	80.4	130	/	20.75	63	22	191	18.59
Day 10	20.86	86.2	248	95.46	/	43	16	/	/
Day 13	14.8	84.9	281	105.47	/	48	26	151	19.87
Day 15	8.55	78.6	260	92.43	0.65	/	/	/	/
Day 18	10.93	81.6	316	50.36	/	/	/	/	/

Normal values of laboratory tests:

White blood cell (WBC): 3.5–9.5 *10^9/L

Neutrophil percentage: 40–75%

Hypersensitive C-reactive protein (hs-CRP): < 10 mg/L

Platelet (PLT): 100–300 *10^9/L

Procalcitonin (PCT): < 0.5 ng/ml

Aspartate aminotransferase (ASL): 0–50 IU/L

Alanine aminotransferase (ALT): 0–50 IU/L

Creatinine: 45–84 µmol/L

Urea nitrogen: 2.1–7.2 mmol/L

## Discussion

*K. pneumoniae* is a species of the Enterobacteriaceae family, which is a normal group of bacteria found in the human mouth and intestines. *K. pneumoniae* includes three subspecies, but usually *K. pneumoniae* refers to the pneumonia subspecies. The main virulence factors of *K. pneumoniae* include capsule serotypes (mainly K1 and K2), hypermucoviscosity phenotype (significantly associated with the syndrome of destructive tissue abscess, including liver or other site abscesses), lipopolysaccharide, ferrisomes, and fimi [9, 10]. Hypervirulent *K. pneumoniae* (hvKp) strains of capsule type K1 and K2 cause invasive infections associated with liver abscesses, which can be difficult to treat and are frequently associated with recurrent infections [11, 12]. Compared with classical *K. pneumoniae*, hvKp has a different iron acquisition capacity, capsule polysaccharide production, phenotype, and biofilm formation, which contribute to its high invasive infection capability [11]. Primary *Klebsiella pneumoniae* liver abscess (KLA) refers to a liver abscess caused by *K. pneumoniae* in the absence of predisposing factors in the abdominal cavity, such as liver and biliary diseases, colorectal diseases, or a history of abdominal surgery or trauma. HvKp is usually the causative agent in these cases [13]. A study of KLA patients in Taiwan, China, showed that 14.1% of K1 strain-infected individuals developed pyogenic endophthalmitis, and 10.5% of K2 strain-infected individuals did so. There were no cases of pyogenic endophthalmitis in non-K1/K2 strain-infected individuals [14]. The infection rate of *K. pneumoniae* increases in patients with compromised primary defense functions, such as diabetes, alcoholism, malignancy, liver and biliary diseases, chronic obstructive pulmonary disease, corticosteroid therapy, and renal failure. These factors may lead to multiple site infections of *K. pneumoniae* [15]. When *K. pneumoniae* causes pulmonary abscess, endophthalmitis, central system infection, necrotic fasciitis, and other multi-site infections, it is clinically referred to as KPIS [16]. A study has shown that the incidence of KPIS caused by hvKp is approximately 12% [10]. Hypermucoviscosity phenotype is present in 98% of invasive strains, and string testing may be one of the screening methods for hvKp. However, it should be noted that 17% of the non-invasive strains also had a hypermucoviscosity phenotype [17].

Diabetes is also an important risk factor for KPIS [18]. The reasons for this are mainly as follows: (1) Elevated blood glucose levels can inhibit the cell activity of CD4, CD8, and NK cells, as well as the production of cytokines, leading to immune dysfunction. In diabetic patients, the initial symptoms of infectious diseases may not be apparent, resulting in delayed treatment [19]. (2) Hyperglycemia inhibits the adhesion, chemotaxis, and phagocytic functions of neutrophils, making infections difficult to control [20]. (3) In diabetic patients, increased blood glucose leads to peripheral vascular disease, increased permeability of the blood vessel wall, and decreased blood oxygen content in the surrounding tissues. These factors provide favorable conditions for bacterial growth and proliferation [21]. Therefore, diabetic patients infected with *K. pneumoniae* are prone to hematogenous dissemination, leading to multiple abscesses in locations such as the intracranial region, lungs, and liver. Strict blood glucose control can prevent the occurrence of metastatic complications, while poor blood glucose control can result in a worse prognosis for patients with KPIS [22–24]. In the majority of critically ill and noncritically ill patients, the target blood glucose control range was 140–180 mg/dL (7.8–10.0 mmol/L). Achieving this goal requires the use of intravenous insulin when the blood glucose level is ≥ 180 mg/dL (10.0 mmol/L) [25]. In the present case, significantly elevated glycosylated hemoglobin upon admission indicated poor recent blood glucose control, which is one of the reasons for the occurrence of KPIS and the poor prognosis of the patient.

For patients with diabetes, especially those with poor blood glucose control, comprehensive examinations should be conducted if KLA occurs. These include chest and abdominal CT scans, as well as cranial MRI, to screen for all possible infection sites. In this case, the patient presented with impaired consciousness, and the head CT scan revealed hypodense lesions. Although the imaging findings lacked specificity, it is still important to remain vigilant for the possibility of intracranial infection. However, at that time, the patient had been intubated and had been undergoing invasive mechanical ventilation, so it was not possible to complete the cranial MRI. It is undeniable that we did not have sufficient awareness of the possibility of brain abscess. These factors contributed to the delayed diagnosis of brain abscess in the patient. Additionally, the patient also had a concurrent fungal infection in the lungs, leading to a very poor prognosis.

The bronchoalveolar lavage fluid, blood, and pus cultures all showed *K. pneumoniae*. After admission, the patient was initially treated with imipenem-cilastatin for anti-infection therapy. Based on the drug sensitivity test and renal function, the antibiotic was switched to piperacillin/tazobactam. Follow-up examinations showed a decrease in infection markers such as WBC, CRP, and PCT. The repeat chest CT scan showed improved absorption of pneumonia, with a reduction in lung nodules and cavities. The repeat abdominal CT scan indicated improvement of the liver abscess. These findings indicate that treatment with piperacillin/tazobactam effectively targeted the infection caused by *K. pneumoniae*. However, the concentration of this drug in the intracranial distribution is low, which may explain the improvement in extracranial infection but the development of brain abscesses. For the selection of antibiotics against KLA, cephalosporins are the main ones at present, which can be combined with aminoglycosides. For intracranial infections, it is important to select antibiotics with high concentrations in the cerebrospinal fluid. The preferred drugs are third-generation cephalosporins, such as ceftriaxone and cefotaxime. In cases where the strain is suspected to produce broad-spectrum β-lactamases, imipenem and meropenem can be administered [24]. Research by Zhang et al. suggests that meropenem is recommended as the first-line treatment for patients with severe infections at multiple sites. However, the duration of antibiotic therapy should be determined based on laboratory and imaging results [2]. For elderly patients, those with diabetes, those with resistance to piperacillin/tazobactam or cefotaxime, patients admitted to the intensive care unit, and other high-risk patients for infection, when diagnosed with KLA, carbapenems should be the preferred choice for antimicrobial treatment [26].

In conclusion, in patients with hvKp infection or concurrent diabetes, caution should be exercised for the occurrence of KPIS when a lung abscess or liver abscess develops. For patients who may have intracranial infection, early completion of cranial MRI and selection of antibiotics with higher distribution in the cerebrospinal fluid are necessary. At the same time, active treatment of the patient’s complications and nutritional support should be provided.

## Data Availability

Not applicable.
